# Expression of cdc25A and cdc25B proteins in thyroid neoplasms

**DOI:** 10.1038/sj.bjc.6600364

**Published:** 2002-06-17

**Authors:** Y Ito, H Yoshida, K Nakano, K Kobayashi, T Yokozawa, K Hirai, F Matsuzuka, N Matsuura, K Kakudo, K Kuma, A Miyauchi

**Affiliations:** Department of Surgery, Kuma Hospital, 8-2-35, Shimoyamate-dori, Chuo-ku, Kobe City 650-0011, Japan; Department of Pathology, School of Allied Health Science, Osaka University Faculty of Medicine, 1-7, Yamadaoka, Suita, Osaka 565-0871, Japan; Department of Pathology, Wakayama Medical College, Kimiidera 811-1, Wakayama City, 641-0012, Japan

**Keywords:** cdc25A, cdc25B, thyroid tumour, immunohistochemistry

## Abstract

Cdc25B and cdc25A phosphates are prominent stimulators of cell cycle progression and recent studies have also suggested their oncogenic roles. To elucidate the role of these proteins in thyroid neoplasms, we immunohistochemically investigated their expression, and neither protein was expressed in normal follicular cells. Cdc25B was frequently overexpressed in follicular adenoma and minimally invasive follicular carcinoma, but the incidence was significantly lower in widely invasive follicular carcinoma. Furthermore, the cdc25B expression level significantly decreased with the dedifferentiation of thyroid carcinoma. Cdc25A overexpression was observed in high incidences in all types of thyroid neoplasms. These results suggest that cdc25B and cdc25A play oncogenic roles in thyroid follicules and that cdc25B works predominantly in the early phase of the progression of thyroid carcinoma, whereas cdc25A plays a fundamental role in the development of thyroid neoplasms.

*British Journal of Cancer* (2002) **86**, 1909–1913. doi:10.1038/sj.bjc.6600364 www.bjcancer.com

© 2002 Cancer Research UK

## 

Thyroid carcinoma is one of the most common malignancies originating from the endocrine organs. Two types of carcinoma are known to occur from normal follicular cells, which are papillary and follicular carcinomas. Follicular carcinoma is said to arise from preexisting follicular adenoma, although this has not yet been confirmed. Precursor lesions of papillary carcinoma, on the other hand, have not yet been identified (Faggin, 2000). It is well known that the biological characteristics of these carcinomas are generally mild and the prognosis is excellent if operated on competently. However, anaplastic carcinoma, also known as undifferentiated carcinoma, arises from papillary or follicular carcinoma and is one of the most aggressive human carcinomas with a dire prognosis. Although many kinds of therapeutic strategy have been performed, most patients die within 6 months after diagnosis (Aldinger *et al*, 1978). Furthermore, Sakamoto *et al* (1983) demonstrated that papillary or follicular carcinoma with a solid, trabecular or scirrhous growth pattern showed a worse clinical outcome than pure papillary or follicular carcinoma. They proposed a clinicopathological entity of poorly differentiated carcinoma, and hypothesised that this type of carcinoma falls between well differentiated carcinoma and undifferentiated carcinoma, although it is still an open question whether showing such growth patterns is truly due to the dedifferentiation of carcinoma.

To evaluate the biological aggressiveness, one prominent factor is the cell proliferating activity, which reflects the cell cycle progression. In the cell cycle, two checkpoints have been identified, which are located in the G1-S phase and the G2-M phase. The complexes of various cyclins and cyclin dependent kinases (CDKs) play a crucial role in exceeding these checkpoints and CDKs should be activated by phosphorylation in order to work as positive regulators of the cell cycle (Sherr, 1994). Two kinds of phosphatase, cdc25A and cdc25B are known to active CDKs, resulting in positive regulation of the cell cycle progression (Galactinov and Beach, 1991). As cdc25A mRNA expression is elevated in the late G1 phase and the microinjection of a specific antibody against cdc25A blocks G1-S transition, it is suggested that cdc25A activates the CDKs, making a complex with the G1 cyclins (Jinno *et al*, 1994). On the other hand, cdc25B plays a crucial role in G2-M transition, of which the target should be cyclin B1/cdc2 complex (Lammer *et al*, 1998). Furthermore, cdc25A and cdc25B can induce a malignant transformation in rodent cells in cooperation with either Ha-RASG12V or loss of RB1, indicating that they are potential oncogenes (Galactinov *et al*, 1995).

The expression of cdc25A and cdc25B has been studied in some human neoplasms and various results have been obtained (Kudo *et al*, 1997; Wu *et al*, 1998; Broggini *et al*, 2000; Takemasa *et al*, 2000; Nishioka *et al*, 2001; Sasaki *et al*, 2001). In thyroid carcinomas, studying the modulation of cell proliferation is also important, because the activity of cell proliferation strongly reflects the biological aggressiveness of this carcinoma (Erickson *et al*, 1998). In this study, therefore, we investigated the expression of cdc25A and cdc25B in order to elucidate the role of these proteins as modulators of the cell cycle progression of this carcinoma.

## MATERIALS AND METHODS

### Tissue specimens

Ten per cent formalin-fixed, paraffin-embedded blocks were prepared from the surgical specimens of 172 cases of thyroid tumours. They consisted of 19 follicular adenomas, 26 minimally invasive follicular carcinomas, 23 widely invasive follicular carcinomas, 72 cases of papillary carcinomas, and 32 cases of anaplastic (undifferentiated) carcinomas. This project was approved by the Ethics Committees of Kuma Hospital.

### Antibodies

Polyclonal antibodies against cdc25B (sc-326) and cdc25A (sc-97) were purchased from Santa Cruz Biotechnology (Santa Cruz, CA, USA). They were applied as primary antibodies at a concentration of 1 : 200 and 1 : 400, respectively.

### Immunohistochemistry

Tissue sections 4 μm thick were dewaxed and endogenous peroxidase activity was blocked with 0.3% hydrogen peroxide in methanol for 15 min. After rinsing in distilled water, the sections were then immersed in 0.03 mol L^−1^ citrate buffer (pH 6.0) and incubated at 95°C for 40 min in a water bath for antigen retrieval. After rinsing in phosphate-buffered saline pH 7.2 (PBS), 10% bovine serum (Wako, Osaka, Japan) was applied for 20 min to block nonspecific reactions. Sections were then incubated with the primary antibody overnight at 4°C. After rinsing in PBS, they were treated with peroxidase-labelled anti-rabbit immunoglobulins (Nichirei, Tokyo, Japan) for 30 min. The peroxidase reaction was visualised by incubating the sections with 0.02% 3,3′-diaminobenzidine tetrahydrochloride in 0.05 M Tris buffer with 0.01% hydrogen peroxide (Nichirei, Tokyo, Japan). The sections were counterstained with haematoxylin. Sections for the negative control were prepared using rabbit immunoglobulins instead of the primary antibody.

### Immunohistochemical evaluation

We regarded the cells as positive for these proteins when their immunoreactivity was clearly observed in their nuclei or cytoplasms, or in both. We classified the cases into four categories according to their positive cell rate as follows: (−), <10%, (+), from 10 to 25%, (++), from 25 to 75%, (+++), >75%. We regarded the cases classified as (++) or (+++) as overexpressing these proteins.

### Statistical analyses

We employed Fischer's exact test for analyses of the relationship between the expression of these proteins and their histological types. *P* values less than 0.05 were regarded as statistically significant.

## RESULTS

### Expression of cdc25B

**Figure 1:**
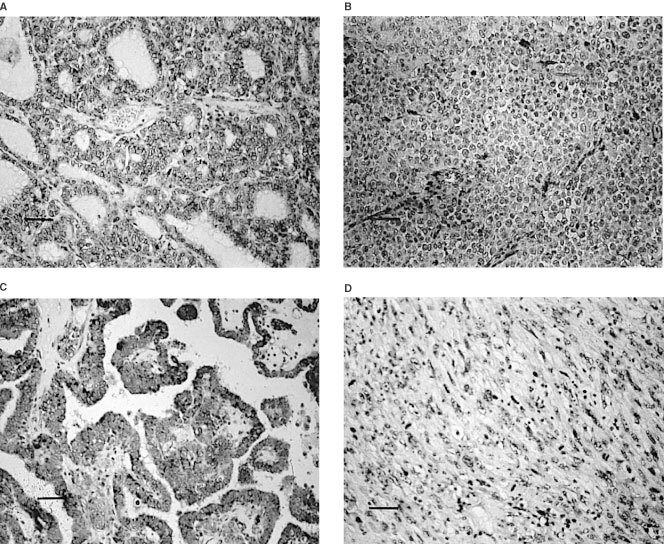
Immunostaining of cdc25B. (**A**) cdc25B overexpression in minimally invasive follicular carcinoma (+++). (**B**) cdc25B overexpression in widely invasive follicular carcinoma with solid growth pattern (++). (**C**) cdc25 overexpression in papillary carcinoma (+++). (**D**) cdc25 was negative in undifferentiated carcinoma (−).

Cdc25B was not expressed in normal follicular cells or stromal cells, including lymphocytes and epithelial cells of the blood vessels (not shown), whereas it was predominantly expressed in the cytoplasms of tumour cells. In follicular tumours, cdc25B overexpression was observed in 41 of the 68 cases (60.3%) (Figure 1A

). In particular, follicular adenoma and minimally invasive follicular carcinoma very frequently overexpressed cdc25B, 63.2% (12 of the 19 cases) and 73.1% (19 of the 26 cases), respectively. In widely invasive follicular carcinoma, this phenomenon was seen in only 43.5% (10 of the 23 cases) of the cases (Figure 1B), which was significantly lower (*P*=0.0456) than that in minimally invasive carcinoma (Table 1

**Table 1:**
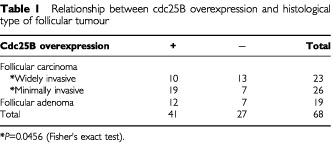
Relationship between cdc25B overexpression and histological type of follicular tumour

).

In papillary carcinoma, 44 of the 72 cases (61.1%) overexpressed cdc25B (Figure 1C), indicating that this phenomenon was observed in 74 of the 121 cases (61.2%) of papillary or follicular carcinoma. Among these cases, 43 had lesions showing a solid, trabecular or scirrhous growth pattern, which was consistent with the criteria of poorly differentiated carcinoma proposed by Sakamoto *et al* (1983) (Figure 1C). Table 2

**Table 2:**
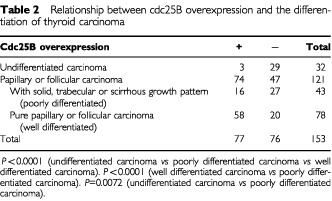
Relationship between cdc25B overexpression and the differentiation of thyroid carcinoma

shows the relationship between cdc25B overexpression and carcinoma differentiation. The incidence of cdc25B overexpression in well differentiated carcinoma was 74.4% (58 of the 78 cases). It was significantly higher than that in poorly differentiated carcinoma (*P*<0.0001), which was 37.2% (16 of the 43 cases). In undifferentiated carcinoma, the incidence was only 9.3% (three of the 32 cases) (Figure 1D), which was lower than in poorly differentiated carcinoma (*P*=0.0072). Thus, the reduced expression of cdc25B was significantly linked to dedifferentiation of thyroid carcinoma (*P*<0.0001).

### Expression of cdc25A

**Figure 2:**
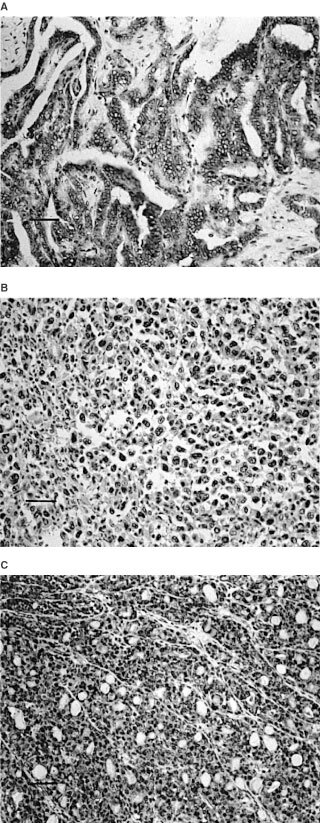
Immunostaining of cdc25A. (**A**) cdc25A overexpression in cytoplasms of papillary carcinoma (+++). (**B**) cdc25A overexpression in cell nuclei of undifferentiated carcinoma (+++). (**C**) cdc25A overexpression in cytoplasms and cell nuclei of follicular adenoma (+++). Scale bars: 150 μm.

Cdc25A expression was not observed in normal follicular cells or stromal cells (not shown). In thyroid neoplasms, its immunoreactivity was localised in the nuclei or cytoplasms or in both. One hundred and five cases of 153 thyroid carcinomas (68.7%) (Figure 2A,B

) and six cases of 19 adenomas (31.6%) overexpressed cdc25A (Figure 2C). Among them, cdc25A was localised predominantly in the cell nuclei in 29 carcinomas (27.6%) (Figure 2B) and three adenomas (50%), in the cytoplasms in 66 carcinomas (62.9%) (Figure 2A) and two adenomas (33.3%), and in the remaining 10 carcinomas (9.5%) and one adenoma (16.7%) (Figure 2C), it was expressed both in the nuclei and cytoplasms in similar incidences. We investigated the relationship between the cell localisation of cdc25A and histological types, but no statistically significant relationships were established (not shown).

We investigated the relationship between cdc25A expression and clinicopathological features, as shown in Tables 3

**Table 3:**
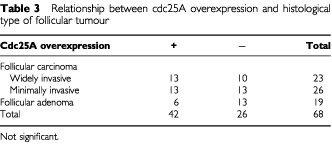
Relationship between cdc25A overexpression and histological type of follicular tumour

and 4

**Table 4:**
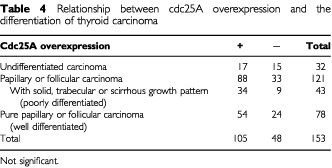
Relationship between cdc25A overexpression and the differentiation of thyroid carcinoma

, although we could not establish any significance. Furthermore, no relationships were determined between the expression of cdc25A and cdc25B in any histological types (data not shown).

## DISCUSSION

In this study, we demonstrated that (1) cdc25B and cdc25A were not overexpressed in normal thyroid tissue, (2) cdc25B was frequently overexpressed in follicular adenoma and minimally invasive follicular carcinoma, but the incidence significantly decreased in widely invasive follicular carcinoma, (3) the incidence of cdc25B overexpression significantly decreased with the dedifferentiation of carcinoma, and (4) cdc25A was frequently overexpressed in thyroid neoplasms regardless of their histological types.

A difference in cell localisation between these two phosphatases has been reported. Previous *in vitro* and clinical studies indicated the cytoplasmic accumulation of cdc25B, which is in agreement with our findings (Gabrielli *et al*, 1996; Gasparotte *et al*, 1997; Kudo *et al*, 1997; Broggini *et al*, 2000; Takemasa *et al*, 2000; Nishioka *et al*, 2001). On the other hand, Dixon *et al* (1998) showed an elevated expression of cdc25A in nuclear fractions of colon carcinoma, which was confirmed by immunohistochemical study (Takemasa *et al*, 2000). However, in oesophageal carcinoma, similar to our results, cdc25A was accumulated both in cytoplasms and nuclei (Nishioka *et al*, 2001), and in ovarian carcinoma, its immunoreactivity was seen mainly in cytoplasms (Broggini *et al*, 2000), indicating that the localisation of this phosphatase depends on the origin of the carcinoma.

What is most interesting in this study is the inverse relationship between cdc25B overexpression and the biological aggressiveness of the thyroid tumour, because such an expression status has not been observed in other positive regulators of cell cycle. Wang *et al* (2000) demonstrated that cyclin D1 was more frequently overexpressed in more aggressive thyroid carcinomas such as anaplastic carcinoma, tall cell variant, and insular carcinoma. On the other hand, Goto *et al* (2001) showed that cyclin D1 was frequently overexpressed in thyroid carcinoma but rarely in benign adenoma. Furthermore, our recent study showed that cyclin A expression level increased with dedifferentiation of thyroid carcinoma and cyclin B1 overexpression was found exclusively in undifferentiated carcinoma (manuscript submitted). It is thus suggested that the reduced expression of cdc25B in dedifferentiated carcinoma is unique compared to other cell cycle regulatory proteins.

The expression status of cdc25B in thyroid tumours differs from that in other carcinomas. Previous studies have demonstrated that cdc25B overexpression reflects a worse clinical outcome in patients with colorectal (Takemasa *et al*, 2000), ovarian (Broggini *et al*, 2000), and non-small-cell lung carcinomas (Sasaki *et al*, 2001). In gastric carcinoma, cdc25B expression was associated with advanced stage and deep invasion (Galactinov *et al*, 1995). However, the physiological roles of cdc25B in carcinomas do not appear to be simple. Takemasa *et al* (2000) demonstrated that, although cdc25B overexpression can be regarded as an independent prognostic factor in colorectal carcinoma, it is not related to the cell proliferating activity evaluated by the Ki-67 labelling index. These findings are strange, because cdc25B fundamentally acts as a positive regulator of cell cycle progression. They thus hypothesised that cdc25B itself displays oncogenic properties by enhancing the malignant nature of this carcinoma. Also, in thyroid carcinoma, the clinical significance of cdc25B overexpression is very complicated. Previous studies have demonstrated that, in thyroid neoplasms, cell proliferating activity is usually low, except for its drastic elevation in undifferentiated carcinoma (Erickson *et al*, 1998). This study showed the frequent overexpression of cdc25B in benign adenoma and carcinomas with low aggressive phenotypes and its decreased expression in those of very aggressive types, such as undifferentiated carcinoma and widely invasive follicular carcinoma, indicating that cdc25B expression is even inversely linked to the cell proliferating activity of thyroid neoplasms. It is thus suggested that cdc25B plays a crucial role in the progression of thyroid carcinoma in the early stage, as well as in the tumorigenesis of follicular cells of the thyroid, rather than merely in tumour cell proliferation. This protein does not seem to be necessary for the development of thyroid carcinoma after it achieves high proliferating activity, including dedifferentiation.

The clinical significance of cdc25A in carcinoma also seems to vary, because different results have been reported for carcinomas of different origin (Broggini *et al*, 2000; Takemasa *et al*, 2000; Nishioka *et al*, 2001). In oesophageal and ovarian carcinomas, its overexpression significantly predicts a poor prognosis whereas no such relation could be established in colorectal carcinoma. According to our findings, cdc25A was frequently overexpressed in all types of thyroid neoplasms including benign adenoma arising from follicular cells. Unlike cdc25B, the incidence of cdc25A overexpression did not decrease in carcinomas with aggressive phenotypes. It is therefore suggested that this phosphatase plays a fundamental role in the oncogenesis of thyroid follicles and also in the development of thyroid neoplasms, regardless of histological type. Previous studies have shown that the overexpression of G1 cyclins could be observed in thyroid neoplasms of various types in similar incidences (Wang *et al*, 2000; Goto *et al*, 2001). Thus, we can hypothesise that cdc25A may be linked to the activation of cdks, making complexes with cyclins, although it is unlikely that both cdc25A and cdk-G1 cyclin complex are directly related to the cell proliferating activity of thyroid neoplasms.

The significant reduction of cdc25B expression in dedifferentiated thyroid carcinoma has prompted researchers to investigate its prognostic value. Of the 32 undifferentiated carcinomas in our series, only 17 cases could undergo curative surgery. Among them, one case which overexpressed cdc25B has survived for 6 months after surgery, whereas the three long term survival cases, 82, 56, and 44 months, did not overexpress cdc25B. We therefore failed to establish the prognostic value of cdc25B expression, but further studies with a larger number of cases undergoing curative surgery are necessary to draw a definitive conclusion about this point.

In summary, this study demonstrated that cdc25B and cdc25A may play an oncogenic role in thyroid neoplasms but may not be directly linked to the cell proliferation of thyroid tumours. Further studies are necessary to more clearly elucidate the significance of these proteins in thyroid neoplasms.
